# Alarming high prevalence of metabolic syndrome among Jordanian adults

**DOI:** 10.12669/pjms.316.7714

**Published:** 2015

**Authors:** Ahmad A. Obeidat, Mousa N. Ahmad, Fares H. Haddad, Firas S. Azzeh

**Affiliations:** 1 Dr. Ahmad Obeidat, PhD. Assistant Professor of Clinical Nutrition, Department of Clinical Nutrition, Faculty of Applied Medical Sciences, Taibah University, Yanbu, Saudi Arabia; 2 Dr. Mousa Ahmad, PhD. Associate Professor of Clinical Nutrition, Department of Nutrition and Dietetics, Faculty of Agriculture, University of Jordan, Amman, Jordan; 3 Dr. Fares Haddad, MD. Endocrinologist, Department of Endocrinology, King Hussein Medical Center, Amman, Jordan; 4 Dr. Firas Azzeh, PhD. Associate Professor of Clinical Nutrition, Dept. of Clinical Nutrition, Faculty of Applied Medical Sciences, Umm Al-Qura University, Makkah, Saudi Arabia

**Keywords:** Metabolic Syndrome, Prevalence, Jordan

## Abstract

**Objective:**

To evaluate the prevalence and the individual components of metabolic syndrome (MetS) in Jordanian adults.

**Methods:**

In this cross sectional study, 630 adult subjects (308 men and 322 women) aged between 20-70 years were recruited from the clinics at the King Hussein Medical Center. The diagnosis of MetS was made according to the International Diabetes Federation (IDF) criteria-2005. Blood samples were collected after 10-12 hours overnight fasting and serum was obtained for biochemical analysis.

**Results:**

The prevalence of metabolic syndrome according to IDF criteria was 51% (46.4% in men and 55.3% in women). Prevalence of increased waist circumference in the total sample was 71.6%, 46% for high blood pressure, 42.4% for elevated fasting blood glucose, 43.5% for low high density lipoprotein, and 50.2% for hypertriglyceridemia.

**Conclusion:**

The prevalence and individual components of MetS in Jordan were high. Screening of MetS is needed at national level to reduce the incidence of Type 2 diabetes mellitus (T2DM) and cardiovascular disease (CVD).

## INTRODUCTION

Metabolic syndrome (MetS) is a huddle of interrelated metabolic risk factors that increase the risk of cardiovascular morbidity and mortality.^1^-^3^ The frequently documented factors include insulin resistance or glucose intolerance, central/abdominal obesity, hypertension and dyslipidemia, particularly decreased high density lipoprotein cholesterol (HDL-C) and hypertriglyceridemia (high blood triglycerides (TG)). These factors are the major risk factors for cardiovascular disease (CVD).^1^,^3^ MetS is essentially considered as a product of interaction between multiple genetic and environmental factors, though its pathogenesis is not clearly determined.^4^

Risk factor clustering in the MetS cannot be explained by chance alone.^5^ Therefore, the syndrome is widely accepted as an important risk factor for CVD, in addition to other environmental/genetic factors such as age, sex, and smoking.^5^ Each component of MetS conveys increased risk of CVD, but the combination of different abnormalities results in a synergistic effect; producing a much greater risk than the sum of the individual components of this syndrome.^6^ As the various abnormalities of MetS may be documented up to 10 years before the detection of type 2 diabetes mellitus (T2DM) or CVD, there is a potential to prevent both of them in persons identified with MetS.^6^ The purpose of identifying people with the MetS is to reduce the long term risk of developing diabetes, CVD, other forms of atherosclerotic disease, chronic renal disease, obstructive sleep apnea, nonalcoholic fatty liver disease, and gout.^7^

MetS is currently thought to be the underlying major cause of diabetes and CVD epidemics worldwide; resulting in premature morbidity and mortality, in addition to increased economic strain on the health systems of most countries.^3^,^7^,^8^ The MetS is also associated with other medical conditions, notably, nonalcoholic fatty liver disease, cholesterol gallstones, gout, obstructive sleep apnea, musculoskeletal disease, polycystic ovarian syndrome, and depression.^5^

The MetS has gained a great interest worldwide because of its increasing prevalence.^6^ The MetS is a very common disorder worldwide, with a prevalence ranging from 14-32%,^9^-^14^ and the prevalence increases with age group for both sexes.^13^,^15^ MetS is reaching epidemic proportions, magnified by western-style diets and sedentary lifestyles. Consequently, the prevalence of T2DM and CVD is also likely to rise.^14^,^15^ The differences in prevalence of MetS depend on population characteristics (such as ethnicity, age and sex), geographic location, and the criteria used for the definition.^10^,^15^-^17^

Based on the National Health and Nutrition Examination Survey (NHANES) reports during the period 1999-2002, it is estimated that 34.6% of the United States (US) population have MetS.^10^ The age-adjusted prevalence of MetS in the US for adults is 23.7 %.^17^ In Sweden,^13^ the prevalence of MetS was 14.8% in men and 15.3% in women; in Italy,^12^ the prevalence of MetS in men was 19.6% and much higher in women (33.3%); in India,^18^ MetS was identified in 18.3% of the study population; and in Iran,^19^ the prevalence of MetS was estimated at 33.2%.

In the Arab population, the prevalence of MetS also differs between countries. In Lebanon,^20^ the overall prevalence of MetS was 31.2% (38.6% in men and 25.8% in women); in Oman,^21^ the age-adjusted prevalence was 19.5% among men and 23.0% among women; in United Arab Emirates (UAE),^22^ the prevalence rate is over 40%; and in the West Bank,^9^ the age-adjusted prevalence of MetS was estimated at only 17%.

In Jordan, several studies have focused on estimating the prevalence of the major risk factors for CVD rather than in the clustering form of MetS and have shown a high prevalence of diabetes mellitus, obesity, hypertension and dyslipidemia.^23^-^27^ Studies showed that the prevalence of obesity was high in adult Jordanians over 25 years of age, about 49.7% (23.7% in men and 59.8% in women).^23^-^27^

However, recent studies on the clustering of the metabolic risk factors in the form of MetS in Jordanian adults are limited. Therefore, the aim of this study was to determine the prevalence as well as the individual components of MetS in Jordanian adults.

## METHODS

This cross-sectional study was carried out at King Hussein Medical Center (KHMC) in Amman, Jordan. In this study, 630 adult healthy subjects (308 men and 322 women) aged between 20-70 years were recruited from the healthy volunteers. Informed consent was obtained from each participant at the start of the study by signing their own information sheets. Ethical Approval was obtained from Royal Medical Services Ethical Committee (Amman, Jordan).

The diagnosis of MetS was made according to the International Diabetes Federation(IDF) criteria.^28^ Subjects were considered to have MetS if waist circumference (WC)(measured at midway between iliac crest and lower ribusing plastic non-strechable tape without clothing) was ≥94 cm for men, and ≥ 80cm for women, plus any two of the following risk factors; 1) triglyceride (TG) ≥150 mg/dL, 2) high density lipoprotein cholesterol (HDL-C)<40 mg/dL for men, and <50 mg/dL for women, 3) blood pressure (BP) ≥130 mmHg systolic BP or ≥85 mmHg diastolic BP, and 4) fasting blood glucose (FBG) ≥100 mg/dL.^28^

Blood pressure was measured by a standard mercury sphygmomanometer (Riester, Germany). Blood samples were collected after 10-12 hours overnight fasting and serum was obtained for biochemical analysis of blood variables by using standard biochemical kits at Princess Iman Center for Laboratory Research and Science (KHMC). The following laboratory measurements were performed and recorded for each subject and their values were taken in subsequent calculations: FBG; TG; and HDL-C.

Statistical analyses were performed using Statistical Program for Social Studies (SPSS), version 20. Results were expressed according to the study needs. Levels of statistical significance were set at P-values of less than 0.05.

## RESULTS

Means and standard error of mean (SEM) of anthropometric and clinical indices by gender are shown in Table-I. The age of the study subjects ranged from 20 to 70 years, with a mean age of 43.26±0.54 years (42.19±0.75 in men and 44.28±0.79 in women). Men had significantly (P<0.05) higher values of weight, height, WC, and TG. On the other hand, women had significantly (P<0.05) higher values of diastolic BP and HDL-C. Age, systolic BP, and FBG were not different (P>0.05) between men and women.

**Table-I:** Age and metabolic syndrome components characteristics by gender.

Indices	Mean ± SEM		
	Men (n=308)	Women (n=322)	Total (n=630)
Age (Years)	42.19 ± 0.75	44.28 ± 0.79	43.26 ± 0.54
Weight (Kg) ***	90.34 ± 1.16	81.30 ± 1.21	85.72 ± 0.86
Height (cm) ***	172.18 ± 0.35	158.89 ± 0.35	165.38 ± 0.36
Waist Circumference (cm) **	101.79 ± 0.83	97.76 ± 1.09	99.73 ± 0.70
Systolic Blood Pressure (mmHg)	132.69 ± 1.73	136.96 ± 1.42	134.87 ± 1.12
Diastolic Blood Pressure(mmHg) ***	79.58 ± 0.68	83.80 ± 0.61	81.74 ± 0.46
Fasting Blood Glucose (mg/dl)	124.30 ± 3.39	119.15 ± 3.25	121.67 ± 2.35
High Density Lipoprotein(mg/dl) ***	46.23 ± 0.75	50.66 ± 0.83	48.50 ± 0.57
Triglycerides (mg/dl) **	172.45 ± 4.47	153.91 ± 4.96	162.97 ± 3.37

** Significant at P-value < 0.01;

*** Significant at P-value < 0.001.

The proportions and numbers of MetS components are shown in Table-II. Prevalence of increased WC in the total sample was 71.6%, while 46%, 42.4%, 43.5%, and 50.2% of the total sample were high in BP, elevated FBG, low in HDL, and having hypertriglyceridemia, respectively. Males were found to be higher in elevated FBG and hypertriglyceridemia than females, whereas other components were lower.

**Table-II:** Prevalence of metabolic syndrome components by gender.

Indices	N / (% of Total)		
	Men (n=308)	Women (n=322)	Total (n=630)
Increased Waist Circumference; (IDF,2005; ≥94 cm in men, ≥80 cm in women)	213 (69.2)	238 (73.9)	451 (71.6)
High Blood Pressure (IDF,2005; ≥ 130/85 mmHg)	105 (34.1)	187(57.6)	292 (46.3)
Elevated Fasting Blood Glucose (IDF,2005; ≥ 100 mg/dl)	133 (43.2)	134 (41.6)	267 (42.4)
Low High Density Lipoprotein (IDF,2005; < 40 mg/dl in men, <50 mg/dl in women)	107 (34.7)	167 (51.9)	274 (43.5)
Hypertriglyceridemia (IDF,2005; ≥ 150 mg/dl)	180 (58.4)	136 (42.2)	316 (50.2)

***Abbreviation***: IDF: International Diabetes Federation.

The prevalence of metabolic syndrome (defined as increased WC plus two or more of the other risk factors according to IDF criteria-2005^28^) in the study group was 51% (46.4% in men and 55.3% in women), as shown in Table-III.

**Table-III:** Prevalence of metabolic syndrome according to IDF criteria by gender.

Metabolic Syndrome	N/(% of Total)		
	Men (n=308)	Women (n=322)	Total (n=630)
Present ***	143 (46.4)	178 (55.3)	321 (51)
Absent	165 (53.6)	144 (44.7)	309 (49)

***Abbreviation***: IDF: International Diabetes Federation.

*** Significant at P-value < 0.001.

The study groups were analyzed with respect to the presence of MetS risk factors; elevated FBG, hypertension, hypertriglyceridemia, and low HDL-C (Table-IV). Obesity risk was excluded because it is a pre-requisite criterion for the definition of MetS according to the IDF criteria^28^ used in this study. Among all subjects included in the study, 22.7% had no risk factors for MetS, 22.5% had only one risk factor, 21.6% had two risk factors, 16% had three risk factors, and 17.1% had all four risk factors, as shown in Table-IV.

**Table-IV:** Number of risk factors of metabolic syndrome after excluding waist circumference by gender.

Present No. of risk factors	N/(% of Total)		
	Men (N=308)	Women (N=322)	Total (N=630)
0	75 (24.4)	68 (21.1)	143 (22.7)
1	80 (26)	62 (19.3)	142 (22.5)
2	60 (19.5)	76 (23.6)	136 (21.6)
3	47 (15.3)	54 (16.8)	101 (16)
4	46 (14.9)	62 (19.3)	108 (17.1)

## DISCUSSION

The metabolic syndrome is associated with a 5-fold increase in the incidence of T2DM and a 2-3 fold increase in the incidence of CVD.^1^,^3^ Thus it’s very crucial to identify those with MetS as early as possible, so that interventions may help to prevent the development of its complications including diabetes and CVD. Limited recent knowledge exists regarding the prevalence of the MetS in Jordan.

The IDF definition^28^ was adopted in this study for identifying subjects with MetS and different risk factors. The prevalence of metabolic syndrome according to IDF criteria in the current study was high in Jordan (51%) with a significantly (P<0.05) higher prevalence in women (55.3%) than in men (46.4%). Many studies suggest that a higher prevalence of MetS is identified using the IDF criteria than the National Cholesterol Education Program *Adult Treatment Panel III (NCEP* ATPIII) criteria, as the IDF definition uses lower cut-off points for waist circumference.^18^,^19^,^29^,^30^ Also, in Tunisia MetS prevalence was 45.5% according to the IDF criteria and 24.3% according to the ATP III criteria, with significantly (P<0.05) higher prevalence in women than in men.^31^ In one study from Jordan at 2007, the prevalence of MetS in adult northern Jordanians using ATP III criteria was 36.3% (28.7% in men and 40.9% in women).^32^ In a another study conducted at 2010 on 345 hypertensive Jordanian patients (143 men and 202 women),^33^ the prevalence of MetS according to the WHO criteria was 26.9%, with almost no differences between men and women. Both ATPIII and IDF criteria identified around 65% of the study population having MetS.^33^ Previous results in Jordan during 2007 and 2014 years could demonstrate that the prevalence of MetS increased with years. In opposite, the prevalence of MetS in adult US population from 1999 to 2010 years decreased from 25.5% to 22.9%,^34^ and prevalence of MetS in females increased significantly (P= 0.005) with age but not in males (P= 0.54).^34^ The sex difference in the prevalence of the metabolic syndrome in this study is similar to that in other studies.^21^,^31^,^32^,^35^,^36^ Binary logistic regression for gender showed that women have about 1.4 higher risk of MetS incidence than males (Odds ratio: 1.426, 95% CI: 1.042-1.952, P-value: 0.027). Women showed a higher risk factor for carotid atherosclerosis thanmen,^29^ and consequently higher CVD and MetS possibility.

In the IDF definition for MetS, central obesity (increased WC) is a pre-requisite criterion in addition to two or more of the other major risk factors.^28^ Prevalence of abdominal obesity expressed as increased WC was the most common abnormality (71.6%) in men (69.2%) and women (73.9%), followed by dyslipidemia (low HDL cholesterol and hypertriglyceridemia). Previous results were in accordance with many researches^21^,^31^,^32^,^36^,^37^ to the effect that the most common abnormality in MetS was related to increased WC, followed by low HDL cholesterol. It is thought that the modern luxurious life style lies behind abdominal obesity and dyslipidemia being the most common components of metabolic syndrome.^36^,^37^ The highest proportions of metabolic syndrome components (Table-II) in women was observed in increased WC (73.9%) and high BP (57.6%), while for men it was related to increased WC (69.2%) and hypertriglyceridemia (58.4%). Chuengsamarn and his colleagues^29^ confirmed the last results who concluded that the appropriate components to predict Mest, as defined by the IDF criterion, were high BP and increased WC in females, as well as high triglyceride in males.

### Limitations of the study

The study was limited by its relatively small sample size and its design.

## CONCLUSION

There is high prevalence of MetS in Jordan. Screening of MetS is needed at national level to reduce the incidence of T2DM and CVD.
